# Relationship between Phylogenetic Distribution and Genomic Features in *Neurospora crassa*

**DOI:** 10.1371/journal.pone.0005286

**Published:** 2009-04-21

**Authors:** Takao Kasuga, Gertrud Mannhaupt, N. Louise Glass

**Affiliations:** 1 Department of Plant and Microbial Biology, University of California, Berkeley, California, United States of America; 2 United States Department of Agriculture - Agricultural Research Service, Department of Plant Pathology, University of California Davis, Davis, California, United States of America; 3 Helmholtz Centre Munich, German Research Centre for Environmental Health (GmbH), Institute of Bioinformatics and Systems Biology, Neuherberg, Germany; Texas A&M University, United States of America

## Abstract

In the post-genome era, insufficient functional annotation of predicted genes greatly restricts the potential of mining genome data. We demonstrate that an evolutionary approach, which is independent of functional annotation, has great potential as a tool for genome analysis. We chose the genome of a model filamentous fungus *Neurospora crassa* as an example. Phylogenetic distribution of each predicted protein coding gene (PCG) in the *N. crassa* genome was used to classify genes into six mutually exclusive lineage specificity (LS) groups, i.e. Eukaryote/Prokaryote-core, Dikarya-core, Ascomycota-core, Pezizomycotina-specific, *N. crassa*-orphans and Others. Functional category analysis revealed that only ∼23% of PCGs in the two most highly lineage-specific grouping, Pezizomycotina-specific and *N. crassa*-orphans, have functional annotation. In contrast, ∼76% of PCGs in the remaining four LS groups have functional annotation. Analysis of chromosomal localization of *N. crassa*-orphan PCGs and genes encoding for secreted proteins showed enrichment in subtelomeric regions. The origin of *N. crassa*-orphans is not known. We found that 11% of *N. crassa*-orphans have paralogous *N. crassa*-orphan genes. Of the paralogous *N. crassa*-orphan gene pairs, 33% were tandemly located in the genome, implying a duplication origin of *N. crassa*-orphan PCGs in the past. LS grouping is thus a useful tool to explore and understand genome organization, evolution and gene function in fungi.

## Introduction

A windfall of fungal genome sequences has been made available in the past ten years; at present, the genome sequences of ∼40 filamentous fungal species are available. The availability of genome data has unquestionably revolutionized fungal research. At the same time, a large proportion of predicted genes in filamentous fungal genomes are annotated as unclassified genes (lacking functional annotation). For example, the first genome that was sequenced from a filamentous fungus was that of an ascomycete species *Neurospora crassa* [1]; 56% of predicted protein coding genes (PCGs) lack functional annotation according to MIPS *Neurospora crassa* DataBase (http://mips.gsf.de/genre/proj/ncrassa/) [2]. This problem prompted us to employ a bioinformatic tool for analysis of the *N. crassa* genome that does not rely on conventional approaches of functional annotation.

Lineage specificity (LS) measures the narrowness of phylogenetic distribution of a gene's homologs in related species [3]. Homologs of highly lineage-specific genes are distributed restrictedly in fewer species in a given phylogeny, while homologs of highly conserved genes are distributed broadly in many groups of species. We determined the LS of each *N. crassa* gene and classified them into six mutually exclusive LS groups using the SIMAP (similarity matrix of proteins) database [4], [5]: (1) Eukaryote/Prokaryote-core (genes with homologs in non-fungal eukaryotes and/or prokaryotes), (2) Dikarya-core (genes with homologs in Basidiomycota and Ascomycota species), (3) Ascomycota-core (4) Pezizomycotina-specific, (5) *N. crassa*-orphan genes and (6) Others (gene homologs identified in prokaryotes or non-fungal eukaryotes in addition to Pezizomycotina, but not in members of the Basidiomycota, Saccharomycotina or Taphrinomycotina).

The phylogenetic distribution of a gene has been suggested to be of biological importance [6]. Recently, we demonstrated a correlation among LS groups to both expression timing during colony development and to the severity of phenotypes upon gene deletion [7]. In order to further advance our knowledge of genome evolution, we examined relationships between LS groups, gene function and chromosomal location. We found *N. crassa*-orphan genes were enriched at subtelomeric regions. It has been proposed that new genes are generated at subtelomeric regions through gene duplication followed by recombination and transposon insertion [8]. In *N. crassa*, however, gene duplication is suppressed by a genome defense mechanism called repeat-induced point mutation (RIP) [9]. RIP has had a profound impact on genome evolution, greatly slowing the creation of new genes through gene duplication [1], [10]. However, in the *N. crassa* genome, 82 pairs of tandemly duplicated *N. crassa*-orphan paralogs were identified. Judging from protein sequence divergence, all the tandemly duplicated *N. crassa-*orphan genes have an ancient origin. Accelerated evolutionary rate of *N. crassa* orphans, rather than gene duplication, seemed to be responsible for the maintenance of the pool of “species-specific” genes, which might play pivotal roles in adaptation and competition of a fungal lineage.

## Results and Discussion

**Figure 1:**
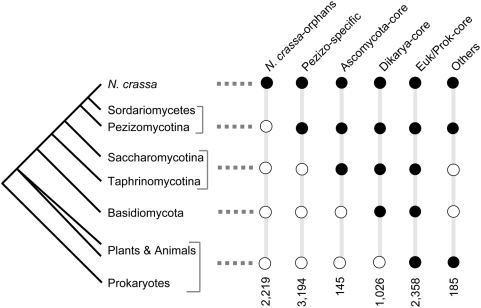
Lineage specificity classification of predicted *N. crassa* protein coding gene (PCG) set based on phylogenetic distribution. A black circle indicates that the gene homolog is present in the corresponding lineage; a white circle means it is absent. Number of PCGs in each LS group is shown at the bottom. Note that *N. crassa* is a member of the class Sordariomycetes, which is within the Pezizomycotina.

**Figure 2:**
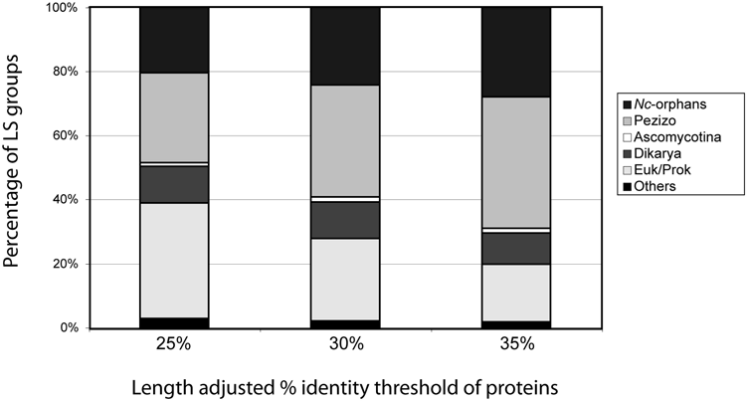
Proportion of lineage specificity (LS) groups in relation to threshold values for length-adjusted percent protein identity. The 9,127 *N. crassa* gene set was classified into five LS groups according to presence/absence of homologs in defined taxonomical units at the given percent protein identity threshold level (25%, 30% or 35%). From the top, the proportion of genes in *N. crassa*-orphan, Pezizo-specific, Ascomycota-core, Dikarya-core, Euk/Prok-core, and Others LS groups are shown. Note that the number of genes in *N. crassa*-orphans and Pezizo-specific groups increases and the threshold level rises from 25% to 35%.

Mutually exclusive LS groups of *N. crassa* PCGs were delimited based on the absence/presence of homologous genes in defined taxonomic units (see also [3]) (Fig. 1, a complete list of genes can be found in Table S1). The membership in each LS group depends of the threshold values for percent protein identity. As anticipated, the higher the threshold value is, the more genes are assigned to specific LS groups such as *N. crassa*-orphan and Pezizomycotina-specific genes (Fig. 2). We therefore chose 30% for the threshold value of length-adjusted protein identity, at which the majority of genes are predicted to encode structurally homologous proteins [11]. Among the phylogenetic groups, 2,358 *N. crassa* PCGs were highly conserved and had at least 30% length-adjusted protein identity with PCGs in non-fungal eukaryotes (e.g. plants and animals) and/or prokaryotes, in addition to Ascomycota and Basidiomycota species. This group of PCGs genes was referred to as Euk/Prok-core. Homologs of 1,026 *N. crassa* PCGs were found in Basidiomycota fungi, in addition to Ascomycota fungi, and which were defined as Dikarya-core genes. Homologs of 145 *N. crassa* PCGs were found in species in the Saccharomycotina (e.g. *Saccharomyces cerevisiae*) and/or Taphrinomycotina (e.g. *Schizosaccharomyces pombe*), but homologs were not identified in non-Ascomycota fungi. This group of PCGs was defined as Ascomycota-core genes. All the Ascomycota-core genes also had homologs in the genomes of Pezizomycotina fungi. Homologs of 3,194 *N. crassa* PCGs were identified in members of the Pezizomycotina, but not in members of the Saccharomycotina or Taphrinomycotina. This group of genes was defined as Pezizo-specific genes. For 2,219 of the 9,127 PCGs predicted in the *N. crassa* genome, homologous genes were not identified in any other genome; these were defined as *N. crassa*-orphans. Of the remaining 185 *N. crassa* genes, at least one homolog was identified in non-fungal eukaryotes or bacteria in addition to Pezizomycotina fungi, but homologous sequences were not identified in the genomes of Basidiomycota, Saccharomycotina or Taphrinomycotina fungi. Since the lineage-specificity and origin of this group of genes was unclear, they were gathered together in a group termed “Others”. The Others group includes genes that are conserved in the Pezizomycotina clade, but which may have been lost or diverged in the genomes of other members of the Ascomycota and Basidiomycota. Others also includes genes that are candidates for horizontally transferred genes (Charles Hall, personal communication). The coverage of sequenced taxa in the database and the quality of annotation influences the membership of LS groups. For instance, the number of genes in the *N. crassa*-orphan group is likely to be reduced upon the release of genomes from closely-related species, such as *N. tetrasperma* and *N. discreta* (currently being sequenced by the Joint Genomes Institute).

### Highly lineage-specific genes evolve faster than more broadly distributed genes

The LS classification is based on a level of protein conservation with respect to phylogenetic profiles. It is not clear what the degree of protein conservation between any two given species is in relation to the LS classification. To address this question, we compared the conservation of orthologous genes between *N. crassa* and the closest relative with a sequenced genome, *Chaetomium globosum*, a member of the Sordariomycetes within the Pezizomycotina clade. In order to identify orthologous PCGs between the two genomes while avoiding misidentifying paralogous gene pairs as orthologous [12], we included *Magnaporthe grisea* (also a Sordariomycete) for comparative genomic searches. PCGs in *N. crassa* and *C. globosum* were called orthologous only when three-species reciprocal blast searches identified genes shared among the three species with the percentage of protein identity between the homologs being higher between *N. crassa* and *C. globosum* as compared to between *M. grisea* and *N. crassa* or *C. globosum*. Among the predicted 11,124, 9,127 and 12,814 PCGs in *C. globosum*, *N. crassa* and *M. grisea*, respectively, the three-way blast search identified 3,382 homologous genes. Of these, 2,458 were identified as orthologous between *N. crassa* and *C. globosum*. Of these 2,458 genes, 34 were member of Others, 896 were members of the Euk/Prok-core, 333 were members of the Dikarya-core, 64 were members of the Ascomycota-core, and 1,081 orthologs were within the Pezizo-specific LS group. By using this approach, 50 of *N. crassa*-orphan genes were also identified as having orthologs in *M. grisea* and *C. globosum*, although the length adjusted percent protein identity for these orthologous pairs was below the threshold value of 30%.

**Figure 3:**
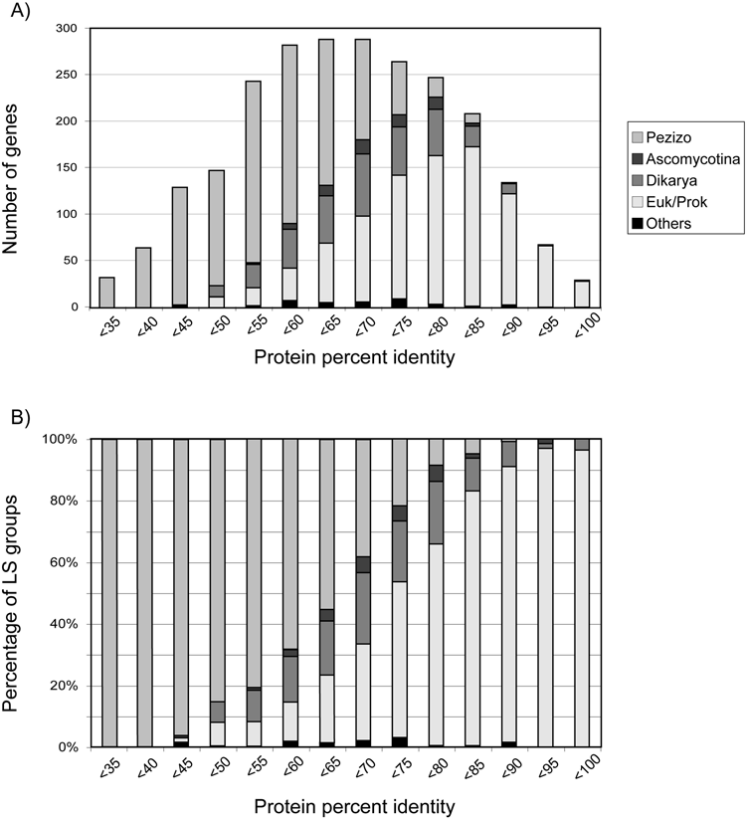
Histograms showing the relationship of percent protein identity scores between *N. crassa* and *C. globosum* and the number (A) and percentage (B) of protein coding genes (PCGs) in each of the lineage specificity (LS) groups. Note that the Pezizo-specific group is larger in the bins of lower protein percent identity as compared to the Euk/Prok-core genes, which is significantly higher in the bins of high protein identity.

The mode of percent identity scores of the entire homologous protein set between *N. crassa* and *C. globosum* lies at the 60–65% bin (Fig. 3A). However, we observed that the mode of percent identity scores for each of the LS groups varied. For example, the Euk/Prok-core group has a mode at 80–85% identity bin, whereas the mode for the Dikarya-core group was lower, at the 65–70% identity bin. Genes within the Pezizo-specific LS group were less conserved, with a mode at the 50–55% identity bin. In fact, genes with protein identity of less than 65% were predominantly Pezizo-specific genes (Fig. 3B). Clearly, there is a correlation between the level of lineage specificity of *N. crassa* genes and the sequence divergence between *N. crassa* and *C. globosum*. A similar trend was observed when percent protein similarity was analyzed instead of percent protein identity (Figure S1). Given that the relative evolutionary rate of a gene can be approximated by sequence divergence between the gene and its homolog, it appears that *N. crassa* genes that are more lineage specific are evolving at a faster rate than those of more broadly distributed genes. This finding is consistent with the observation of elevated nonsynonymous substitution rates within higher lineage specificity groups, such as comparisons between an *Aspergillus fumigatus* and *A. nidulans* pair and a *Saccharomyces cerevisiae* and *S. mikatae* pair [3], which shows a linear relationship between nonsynonymous substitutions and amino acid substitutions [13]. As suggested by Cai et al., [3], genes in a species-specific LS group (such as *N. crassa*-orphan genes) evolve fastest so that their homologs are no longer identifiable in the genomes of related species (such as *C. globosum*).

### Enrichment analysis reveals uneven distribution of functional categories across LS groups

The phylogenetic distribution of a gene is suggested to be biologically important [6]. Genes with the same phylogenetic distribution may have linked functions [14]–[16]. However, the correlation between LS group and gene function has not been systematically investigated. We therefore examined the predicted functions of genes in each LS group using the Functional Catalogue (FunCat) developed by MIPS [17], [18]. It became clear that the vast majority of genes in *N. crassa* orphan group (94%) and Pezizo-specific group (66%) were unclassified (Table 1). In fact, 82% of the total unclassified genes in *N. crassa* belong to either the *N. crassa*-orphan or Pezizo-specific groups. On the other hand, a much smaller portion (23% on average) of Ascomycota-core, Dikarya-core, and Euk/Prok-core genes were unclassified. This result is partly attributable to the extensive functional studies of genes in eukaryotic model organisms, especially in *S. cerevisiae* and *S. pombe*; a majority of Dikarya-core and Euk/Prok-core genes have homologs in *S. pombe* and/or *S. cerevisiae*. In addition to “99 Unclassified proteins”, another example of a functional category that was enriched in the Pezizo-specific group of genes include genes for “11.02.03.04 transcriptional control”. Many fungal-specific transcription factors involved in nutritional metabolism and fungal morphogenesis were in this category. In addition, genes within the categories for “32.05 disease, virulence and defense” and “32.07.01 detoxification involving cytochrome P450” were enriched (Table 2, a full set of enrichment analysis can be found in Table S2). These enriched functional categories reflect experimental studies specific for Pezizomycotina fungi. Of the *N. crassa* orphan genes, only 6.1% have assigned functional categories, and no functional category showed enrichment.

**Table 1:** Functional annotation status of *N. crassa* genes in LS groups

LS group	Annotated	Not annotated	Total
Eukaryote/Prokaryote-core	1968 (83.5%)	390 (16.5%)	2358
Dikarya-core	648 (63.2%)	378 (36.8%)	1026
Ascomycota-core	99 (68.3%)	46 (31.7%)	145
Pezizomycotina-specific	1084 (33.9%)	2110 (66.1%)	3194
*N. crassa*-orphans	136 (6.1%)	2083 (93.9%)	2219
Others	99 (53.5%)	86 (46.5%)	185
Total	4034 (44.2%)	5093 (55.8%)	9127

**Table 2:** Enriched functional categories in each of the six LS-groups

Enriched FunCat categories	Obs. or exp.^a^	*Nc*-orphans	Pezizo-specific	Asco-core	Dikarya-core	Euk/Prok-core	Others	Total^b^
01 METABOLISM	Obs	23	216	16	210∧∧∧	702∧∧∧	61∧∧∧	1228
	Exp	298.6	429.7	19.5	138.0	317.3	24.9	
01.02 nitrogen, sulfur and selenium metabolism	Obs	0	13	1	14	41∧∧∧	7∧∧	76
	Exp	18.5	26.6	1.2	8.5	19.6	1.5	
01.05 C-compound and carbohydrate metabolism	Obs	13	103	7	84∧∧∧	257∧∧∧	32∧∧∧	496
	Exp	120.6	173.6	7.9	55.8	128.1	10.1	
01.05.02.07 sugar, glucoside, polyol and carboxylate catabolism	Obs	0	9	0	5	11	4∧	29
	Exp	7.1	10.1	0.5	3.3	7.5	0.6	
01.05.03 polysaccharide metabolism	Obs	2	29	1	29∧∧∧	37	11∧∧∧	109
	Exp	26.5	38.1	1.7	12.3	28.2	2.2	
01.05.11.07.01 aerobic aromate catabolism	Obs	0	6	0	4	9	4∧∧	23
	Exp	5.6	8.0	0.4	2.6	5.9	0.5	
02 ENERGY	Obs	1	14	2	22	151∧∧∧	3	193
	Exp	46.9	67.5	3.1	21.7	49.9	3.9	
10 CELL CYCLE AND DNA PROCESSING	Obs	6	142	16∧∧	56	163∧∧∧	6	389
	Exp	94.6	136.1	6.2	43.7	100.5	7.9	
11 TRANSCRIPTION	Obs	13	217∧∧∧	12	56	199∧∧∧	4	501
	Exp	121.8	175.3	8.0	56.3	129.4	10.2	
11.02 RNA synthesis	Obs	11	185∧∧∧	9	39	111	3	358
	Exp	87.0	125.3	5.7	40.2	92.5	7.3	
11.02.03 mRNA synthesis	Obs	11	167∧∧∧	8	33	90	3	312
	Exp	75.9	109.2	5.0	35.1	80.6	6.3	
11.02.03.04 transcriptional control	Obs	10	140∧∧∧	6	25	37	2	220
	Exp	53.5	77.0	3.5	24.7	56.8	4.5	
11.02.03.04.01 transcription activation	Obs	1	19∧∧∧	0	1	0	1	22
	Exp	5.3	7.7	0.3	2.5	5.7	0.4	
12 PROTEIN SYNTHESIS	Obs	1	36	10∧	16	197∧∧∧	4	264
	Exp	64.2	92.4	4.2	29.7	68.2	5.4	
14 PROTEIN FATE (folding, modification, destination)	Obs	14	144	18∧	93∧∧	308∧∧∧	10	587
	Exp	142.7	205.4	9.3	66.0	151.7	11.9	
14.07.02 modification with sugar residues (e.g. glycosylation, deglycosylation)	Obs	0	4	4∧∧	7	11	1	27
	Exp	6.6	9.4	0.4	3.0	7.0	0.5	
20 CELLULAR TRANSPORT, TRANSPORT FACILITIES AND TRANSPORT ROUTES	Obs	14	130	17	169∧∧∧	293∧∧∧	9	632
	Exp	153.7	221.2	10.0	71.0	163.3	12.8	
20.01.01.01.01 heavy metal ion transport (Cu+, Fe3+, etc.)	Obs	0	5	2	11∧∧∧	9	1	28
	Exp	6.8	9.8	0.4	3.1	7.2	0.6	
20.01.03 C-compound and carbohydrate transport	Obs	0	12	0	40∧∧∧	22	1	75
	Exp	18.2	26.2	1.2	8.4	19.4	1.5	
20.01.07 amino acid/amino acid derivatives transport	Obs	0	2	0	12∧∧∧	9	0	23
	Exp	5.6	8.0	0.4	2.6	5.9	0.5	
20.09.16 cellular export and secretion	Obs	3	19	4	21∧∧∧	15	1	63
	Exp	15.3	22.0	1.0	7.1	16.3	1.3	
20.09.18 cellular import	Obs	0	15	3	45∧∧∧	29	1	93
	Exp	22.6	32.5	1.5	10.5	24.0	1.9	
20.09.18.07 non-vesicular cellular import	Obs	0	8	2	29∧∧∧	16	0	55
	Exp	13.4	19.2	0.9	6.2	14.2	1.1	
32.05 disease, virulence and defense	Obs	9	55∧	2	25∧∧	23	4	118
	Exp	28.7	41.3	1.9	13.3	30.5	2.4	
32.07 detoxification	Obs	3	31	2	26∧∧∧	30	4	96
	Exp	23.3	33.6	1.5	10.8	24.8	1.9	
32.07.01 detoxification involving cytochrome P450	Obs	0	11∧∧	0	2	0	0	13
	Exp	3.2	4.5	0.2	1.5	3.4	0.3	
43.01.03.05 budding, cell polarity and filament formation	Obs	0	18	6∧∧	9	23∧	0	56
	Exp	13.6	19.6	0.9	6.3	14.5	1.1	
99 UNCLASSIFIED PROTEINS	Obs	2083∧∧∧	2110∧∧∧	46	378	390	86	5093
	Exp	1238.2	1782.3	80.9	572.5	1315.8	103.2	
Total^c^		2219	3194	145	1026	2358	185	9127

a Observed number of genes and expected number of genes if probabilities of each outcome are independent of the LS group.

b Number of genes in each of the functional categories.

c Number of genes in each of the LS groups.

∧ p<0.05, ∧∧ p<0.01, ∧∧∧ p<0.001. p-values due to Fisher's exact test with Benjamini & Hochberg multiple testing correction.

Gene members in the Euk/Prok-core group encode highly conserved PCGs. A large number of functional categories in this group were overrepresented due to the relative paucity of classified genes in the Pezizo-specific and *N. crassa*-orphan LS groups. Some examples of enriched functional categories (p<1×10^−10^) in this group included the main categories “01 Metabolism”, “02 Energy”, “10 Cell cycle and DNA processing”, “12 Protein synthesis”, and “14 Protein fate” (Table 2). Generally, under/over-representation of functional categories in Dikarya-core, and Euk/Prok-core group followed the same trend. However, genes for “01.05.03 polysaccharide metabolism”, “20.01.03 C-compound and carbohydrate transport”, “20.09.16 cellular export and secretion”, “20.09.18.07 non-vesicular cellular import”, and “32.07 detoxification”, were specifically enriched in the Dikarya-core group. The majority of fungi that have been sequenced within the Dikarya-core group have association with plants, either as pathogens, saprophytes or in symbiotic relationships. Thus, an enrichment of genes within this category may reflect the ecological niche of these fungi. There were only 145 genes belonging to Ascomycota-core. Enriched groups of genes peculiar to this group were “14.07.02 modification with sugar residues”, “43.01.03.05 budding, cell polarity and filament formation”. The group “Others” was comprised of 185 genes that showed enrichment for “01.02 nitrogen, sulfur and selenium metabolism”, “01.05.02.07 sugar, glucoside, polyol and carboxylate catabolism”, “01.05.03 polysaccharide metabolism” and “01.05.11.07.01 aerobic aromate catabolism”. Outside of the Pezizomycotina, most of the genes in these categories showed the highest homology to genes in bacterial species.

### Origin of orphan genes in *N. crassa*

**Figure 4:**
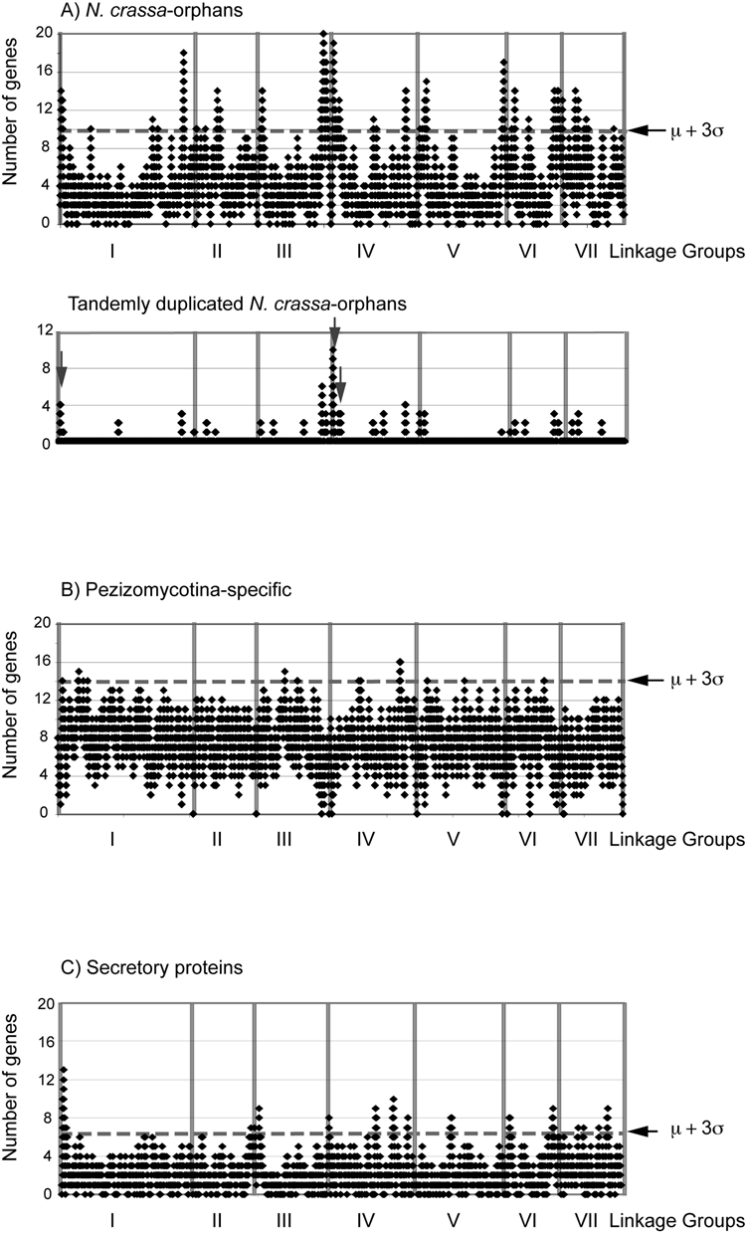
Distribution of A) *N. crassa*-orphan, B) Pezizomycotina-specific and C) secretory PCGs across the seven chromosomes. Distribution of these gene sets were evaluated for every 20 PCGs along *N. crassa* chromosomes I to VII. The chromosomal distribution of tandemly duplicated *N. crassa*-orphan PCGs is shown at the lower panel in A). The vertical grey arrows indicate clusters of five or more gene paralogs. Boundaries between chromosomes are shown with vertical lines. A mean density+3 standard deviations (p = 0.001) according to binomial distribution is shown with a horizontal black arrow in each panel.

A substantial fraction of putative genes in genomes are found to lack sequence similarity to any of the genes in public databases [19], [20]. The origin of these “species-specific genes” or “orphan genes” is not well understood. However, an enrichment of orphan genes has been found at subtelomeric regions in various eukaryotes such as the malaria parasite, *Plasmodium falciparum* [21], [22], in hemiascomycete yeasts [23] and in *Aspergillus* species [24]. We therefore mapped the chromosomal localization of orphan PCGs in *N. crassa* to evaluate the link between the generation of new genes and subtelomeric regions. An uneven distribution of *N. crassa*-orphan genes toward subtelomeric regions was apparent for linkage group I, III, IV, V and VI (Fig. 4A). Uneven distribution of other LS groups was not observed (e.g. Pezizo-specific genes, Fig. 4B).

Because *N. crassa*-orphans were found concentrated at subtelomeric regions, these locations may be sites where *N. crassa*-orphans are generated. Gene duplication is thought to have a primary role in the innovation of new genes [25] and subtelomeric regions are often hotspots for de novo gene duplications [26], [27] or they host duplicated genes that originated in another part of the genome [8]. Thus, it is possible that duplicated genes at subtelomeric regions may serve as a source for species-specific orphan PCGs. We therefore evaluated whether gene duplication at subtelomeric regions was associated with *N. crassa* orphan PCGs. We used a relaxed stringency for protein homology search without length adjusted % protein identity threshold value due to the possible divergence of gene duplications in *N. crassa* due to RIP [9]. A sequence similarity search showed that 385 out of 2,219 *N. crassa* orphan PCGs had homologous sequences, or paralogs, in the *N. crassa* genome (Table S1). Of the 385 best-hit homologous genes, 250 genes belonged exclusively to *N. crassa*-orphan group. The remaining *N. crassa* 135 orphan genes showed some similarity to genes in other LS groups, albeit at <30% length adjusted similarity. We then examined the relative location of best-hit paralogous pairs; the 250 *N. crassa*-orphan and *N. crassa*-orphan (Orph:Orph) pairs and the 135 *N. crassa*-orphan:non-*N. crassa*-orphan (Orph:Non) pairs. Of the 250 Orph:Orph paralogous gene pairs, 82 (33%) were adjacent to each other in the genome. The probability of a pair of genes located adjacent to each other in the *N. crassa* genome is very low (p = 2×10^−4^), emphasizing the biological relevance of the occurrence of consecutive paralogous gene pairs. In contrast, only 6 out of the 135 Orph:Non paralogous gene pairs (4%) were adjacent to each other. As we hypothesized, the highest occurrence of duplicated gene pairs was identified in subtelomeric regions (Fig. 4-A lower panel, linkage groups I, III and IV). In the genome, gene families comprised of *N. crassa*-orphans were identified, and clusters of such paralogous genes were also located at the subtelomeric regions (arrows, Fig. 4A, lower panel).

**Figure 5:**
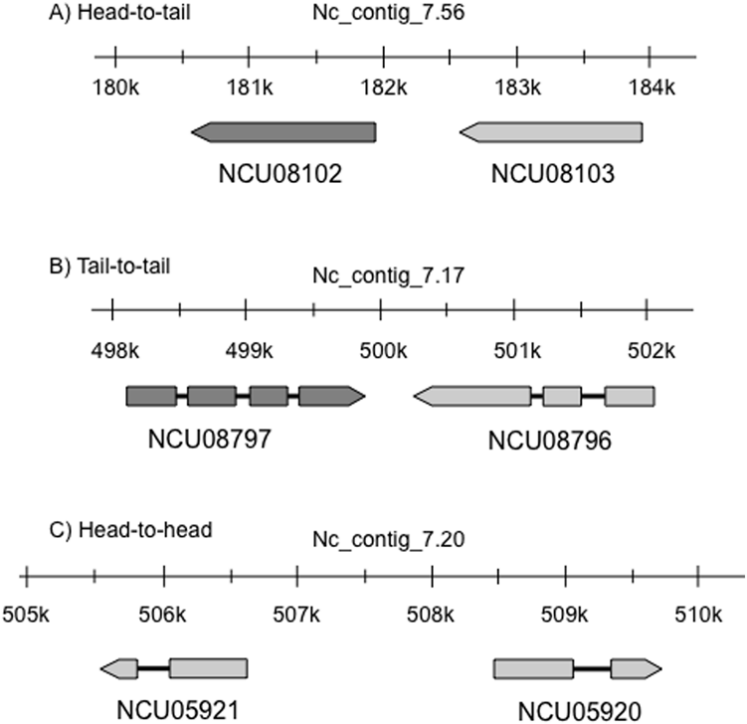
Examples of gene organization of tandem paralogous pairs of *N. crassa*-orphan PCGs. A) a paralogous gene pair, NCU08102 and NCU08103, shows a head-to-tail gene organization; 73 paralogous PCG pairs have this conformation. B) NCU08797 and NCU08796 are one of five cases of paralogous gene pairs having a head-to-head gene organization. C) NCU05920 and NCU05921 are one of four pairs of paralogous genes that show a tail-to-tail gene organization. Schematic representations are derived from the MIPS *Neurospora crassa* DataBase (http://mips.gsf.de/genre/proj/ncrassa/).

We then examined the relative orientation of consecutive paralogous gene pairs. Of the 82 Orph:Orph paralogous duplication PCG pairs, 73 showed the head-to-tail conformation, while 4 of the paralogous duplication pairs showed a tail-to-tail and 5 had a head-to-head organization (Fig. 5). In the entire *N. crassa* genome, only 44% of neighboring PCGs have a head-to-tail organization. Given the genome statistics, the head-to-tail organization was enriched in Orph:Orph paralogous PCG duplication pairs (p = 1.6×10^−17^ by binomial probability). Likewise, although, there were only 6 Orph:Non paralogous consecutive pairs in the genome, 4 of them showed a head-to-tail conformation. Taken together, this enrichment of consecutive paralogous PCG pairs and their head-to-tail organization indicate involvement of tandem duplication events for the generation of *N. crassa*-orphan genes.

In *N. crassa*, gene duplication is suppressed by a genome defense mechanism called RIP [9]. RIP introduces G:C to A:T mutations very efficiently into duplicated gene copies, especially tandem duplications [28]. Thus, the phenomenon of RIP in *N. crassa* argues against the generation of tandem gene duplications for evolving new gene functions. Because RIP is biased toward CpA/TpG sites, the frequency of RIP within the genome can be evaluated (the RIP index) [1], [29]. Consistent with a full genome survey [10], we failed to detect traces of mutations due to RIP in the duplicated *N. crassa*-orphan PCGs. It is noteworthy that the tandemly duplicated PCG pairs were very distantly related to each other: the mean amino acid identity was only 36.1%, just as low as that observed for the 250 Orph:Orph paralogous gene pair group (35.8%).

These lines of evidence suggest that in *N. crassa*, orphan PCGs were generated through tandem duplications, which likely occurred at subtelomeric regions. However, divergence of these tandemly duplicated genes apparently was not a consequence of RIP. It is possible that the contribution of gene duplication for evolving new gene function may have completely ceased after the severe form of RIP arose in the *Neurospora* lineage subsequent to the split with the *Magnaporthe* lineage (C. A. Cuomo et al., unpublished, presented in [10]). In fact, it has been demonstrated that *N. crassa* possesses many fewer genes in multigene families as compared with other analyzed eukaryotic genomes [1]. Cai and coworkers deduced that orphan genes evolve rapidly through accelerated base substitution rate; similarity to other genes cannot be traced after a certain evolutionary distance [3]. Through a comparative genomic analysis between *N. crassa* and *C. globosum*, we also observed that highly lineage-specific genes evolve faster (Fig. 3). Although recent gene duplications may not be involved in generating new genes in *N. crassa*, the *N. crassa*-orphan PCGs, many of which were created through tandem gene duplications in the past, evolve faster through accelerated base substitution rate and thus may contribute to niche adaptation and competition.

### Uneven distribution of protein coding genes encoding secreted proteins

The functions of *N. crassa*-orphan PCGs are mostly unknown. In plant pathogens, PCG encoding secreted proteins are among the most fast evolving gene set and often form species-specific gene families that have arisen by gene expansion [30]. In the plant pathogenic fungus *Fusarium graminearum*, subtelomeric localization of secretory PCGs has been reported [31]. In contrast, the evolutionary relevance of secretory PCGs in non-pathogenic fungi, such as *N. crassa*, is unknown. These observations motivated us to also examine the chromosomal localization of predicted secretory PCGs in *N. crassa*. In the *N. crassa* genome, 984 proteins that are predicted to be secreted were bioinformatically predicted [32] (Table S1). A recent expansion of secretory PCGs was not identified; both the *N. crassa*-orphan group and the total genome contain c.a. 10% predicted secreted PCGs. In the *N. crassa* genome, it was found that genes for secreted proteins are concentrated at several locations. Especially, as in plant pathogenic fungi, subtelomeric localization of secretory proteins were observed at the ends of linkage groups I, III, IV and VI (Fig. 4-C). *N. crassa* is a saprobe that lives on burnt plant material from which congener species [33], as well as many other fungal and bacterial species are often co-isolated. Secretory proteins concentrated at the subtelomeric regions may thus constitute fast evolving genes perhaps involved in competition against antagonists.

### Conclusion

We recently demonstrated a correlation among LS groups, expression timing during colony development, and severity of mutant phenotypes [7]. In this study, we show LS grouping can be associated with genomic localization and evolutionary history. Thus, LS grouping is a powerful tool in the genomic toolkit for functional genomic studies, a method applicable to any organism with a genome sequence. Potential correlations between LS groups and evolving metabolic pathways are intriguing. In addition, LS grouping can be utilized for identification of antifungal drug targets. For example, genes belonging to Dikarya-core LS group could be potential candidates for drug development for treatment of mycoses caused by ascomycetous and basidiomycetous fungi. As has been shown, defects in Dikarya-core genes are more likely to impair growth of fungi [7], yet these homologs are lacking in the mammalian host genomes. Furthermore, a slower rate of DNA substitutions in Dikarya-core gene suggests that mutations in a drug target gene, which might be attributed to drug resistance phenotypes, are less likely to occur in Dikarya-core genes than, for example, genes in a more lineage specific category.

## Materials and Methods

### Lineage specific grouping

To define the phylogenetic distribution of a gene, the SIMAP (similarity matrix of proteins) database developed by MIPS (http://mips.gsf.de/simap/) was used, which is based on a Smith-Waterman pair-wise comparison of available predicted protein-coding sequences [4], [5]. First, all low complexity regions in the protein sequences were masked and then FASTA calculation was conducted. Each FASTA hit was recalculated without low complexity filtering using the Smith-Waterman algorithm and final Smith-Waterman Scores ≥80 were stored in the database. The length dependency of hits was compensated using a value (identity×overlap / protein length), and threshold values of 25, 30, and 35% were evaluated. We retrieved homologous proteins in hierarchical taxonomic units for the MIPS-curated 9,127 PCGs of *N. crassa* using Taxonomy Search tool at the MIPS *Neurospora crassa* Database (http://mips.gsf.de/genre/proj/ncrassa/Search/Gise/taxonomySearch.html). A phylogenetic profile [3], [15] for each of the *N. crassa* genes was constructed due to presence or absence of homologous sequences in the taxonomic units, i.e. prokaryotes and/or non-fungal eukaryotes (command for 30% length-adjusted threshold: >30[TPE]>30[TPL] OR >30[TPE]>30[TME] OR >30[TPE]>30[TBA]), Basidiomycota (>30[TPE]>30[TBS]), Saccharomycotina and/or Taphrinomycotina (>30[TSZ] OR >30[THE]), and Pezizomycotina (>30[TPE]). The genes were then classified into the mutually exclusive LS groups, Euk/Prok-core, Dikarya-core, Ascomycota-core, Pezizomycotina-specific, *N. crassa*-orphans and Others (remainders) (see Fig. 1, a phylogenetic profile for each of the genes is listed in Table S1). In order to quality-control the retrieved dataset, genomes of *Chaetomium globosum* (http://www.broad.mit.edu/science/data#), *Saccharomyces cerevisiae* (http://downloads.yeastgenome.org/sequence/), *Phanerochaete chrysosporium* (http://genome.jgi-psf.org/Phchr1/Phchr1.home.html), *Drosophila melanogaster* (http://www.fruitfly.org/sequence/download.html) and *Arabidopsis thaliana* (http://www.arabidopsis.org/download/index.jsp), were downloaded as a representative of the Sordariomycetes, Hemiascomycetes, Basidiomycetes, animals, and plants, respectively. Homologous sequences of *N. crassa* PCGs were searched in these genomes using the same criteria used for SIMAP database, and results were subsequently cross-examined with retrieved data from SIMAP. No more than 1% of the SIMAP data and our homology search data showed discrepancies in terms of LS groupings.

### Ortholog and paralog assignments

Protein sequences for the *N. crassa*, *C. globosum* and *Magnaporthe grisea* genomes are downloaded from MIPS (ftp://ftpmips.gsf.de/neurospora/) and The Broad Institute (http://www.broad.mit.edu/science/data#). A 3-way reciprocal BLASTP was used to search for orthologous genes between *N. crassa* and *C. globosum*. Prior to BLASTP, low complexity regions in protein sequences were masked using SEG [34]. Homologous best-hit proteins from the three species were then aligned by ClustalW [35]. Proteins were judged as orthologous if percent protein identity between *N. crassa* and *C. globosum* was higher than that between *N. crassa* and *M. grisea* as well as that between *C. globosum* and *M. grisea*. Orthologous protein sequences of *N. crassa* and *C. globosum* were then aligned using LALIGN in the FASTA package [36], [37], and % identity and % similarity scores were obtained.

In order to search for duplicated genes (paralogs) in the *N. crassa* genome, each of the *N. crassa* protein sequences were aligned against translated *N. crassa* genome using BLASTP. For the search for orthologous and paralogous genes, a BLAST expectation (E) value of 1×10^−10^ was used as cutoff. Clusters of paralogs were identified by BLASTCLUST program (BLAST Basic Local Alignment Search Tool, http://www.ncbi.nlm.nih.gov/Ftp/) [38] used with the following parameters: -S (Blast score identity) 0.5, -L (minimum length coverage) 0.5 for protein sequences.

### Enrichment analysis for functional categories

Nine thousand, one hundred twenty-seven of the *N. crassa* PCGs are curated by MIPS. The Functional Catalogue (FunCat) annotation scheme [17], [18] was used to group genes according to their cellular or molecular functions. Over- or under- representation of functional categories across LS groups was evaluated against an expected hypergeometric distribution using Fisher's exact test in the statistical software R 2.6 (http://bioconductor.org). A significant level of 0.05 was used with a multiple testing correction according to Benjamini and Hochberg [39].

### Mapping of chromosomal localization of LS groups

The physical map and assembly of contigs from the *N. crassa* genome project (http://www.broad.mit.edu/annotation/genome/Neurospora/Home.html) have been aligned with the genetic map of the seven linkage groups (chromosomes) (http://www.bioinf.leeds.ac.uk/~gen6ar/newgenelist/genes/index.html). We examined the gene density of each LS group for every 20 gene models along the seven linkage groups. Physical distances between neighboring genes can vary with chromosomal location, however, this aspect was not taken into account for this analysis.

### Prediction of genes coding for secretory proteins

The 9,127 PCGs were classified according to presence or absence of the secretory signal peptide [32] using SignalP3.0 software (http://www.cbs.dtu.dk/services/SignalP/); 984 genes encoding putative secreted proteins were identified in the *N. crassa* genome (Table S1).

## Supporting Information

Table S1 *Neurospora crassa* genome information. *N. crassa* gene IDs, aliases, hyperlinks to MIPS database, MIPS descriptions, FunCat categories, Pfam domains, SignalP results, and LS classification for each of the % id threshold values are shown. Paralogous genes for *N. crassa*-orphans and tandemly located *N. crassa*-orphans paralogs are also indicated. Codes for LS groups are: N_Evol, F_Evol, A_Evol, B_Evol, E_Evol, and O_Evol for *N. crassa*-orphans, Pezizo-specific, Ascomycota-core, Dikarya-core, Euk/Prok-core and Others, respectively. SIMAP matrices for 25%, 30% and 35% threshold values are also listed in the attached worksheets. In the matrices, presence or absence of homologous sequences of *N. crassa* genes in non-fungal Eukaryotes and/or Prokaryotes (Euk/Prok), Basidiomycota, Saccharomycotina and/or Taphrinomycotina (Asco), Pezizomycotina and *C. globosum* (CHG) are number coded as 1/0, 2/0, 4/0, 8/0 and 0.5/0, respectively, and the total scores were used to define the LS groups. Length-adjusted protein percent identity values of *N. crassa* genes against *Chaetomium globosum* (NC_CHG), *Saccharomyces cerevisiae* (NC_SC), *Phanerochaete chrysosporium* (NC_PhC), *Drosophila melanogaster* (NC_DM) and *Arabidopsis thaliana* (NC_AT) were used to revise the SIMAP derived LS classification.
(9.36 MB XLS)

Table S2 Enrichment analysis of FunCat categories in LS groups. For each FunCat category, a statistical significance according to Fisher's exact test is indicated if multiple hypotheses corrected p<0.05.
(0.17 MB XLS)

Figure S1 Histograms showing the relationship of percent similarity scores between *N. crassa* and *C. globosum* and the number (A) and percentage (B) of PCGs in each of the LS groups. Percent identity scores for number (C) and percentage (D) of PCGs are also shown for comparison. Note that although similarity scores are higher than identity scores, the trend is comparable.
(18.59 MB TIF)
