# Supplementary figures and images for: Relationship between Phylogenetic Distribution and Genomic Features in Neurospora crassa
Source: PLoS One. 2009 Apr 21;4(4):e5286. doi: 10.1371/journal.pone.0005286 (PMC2684829; doi:10.1371/journal.pone.0005286)

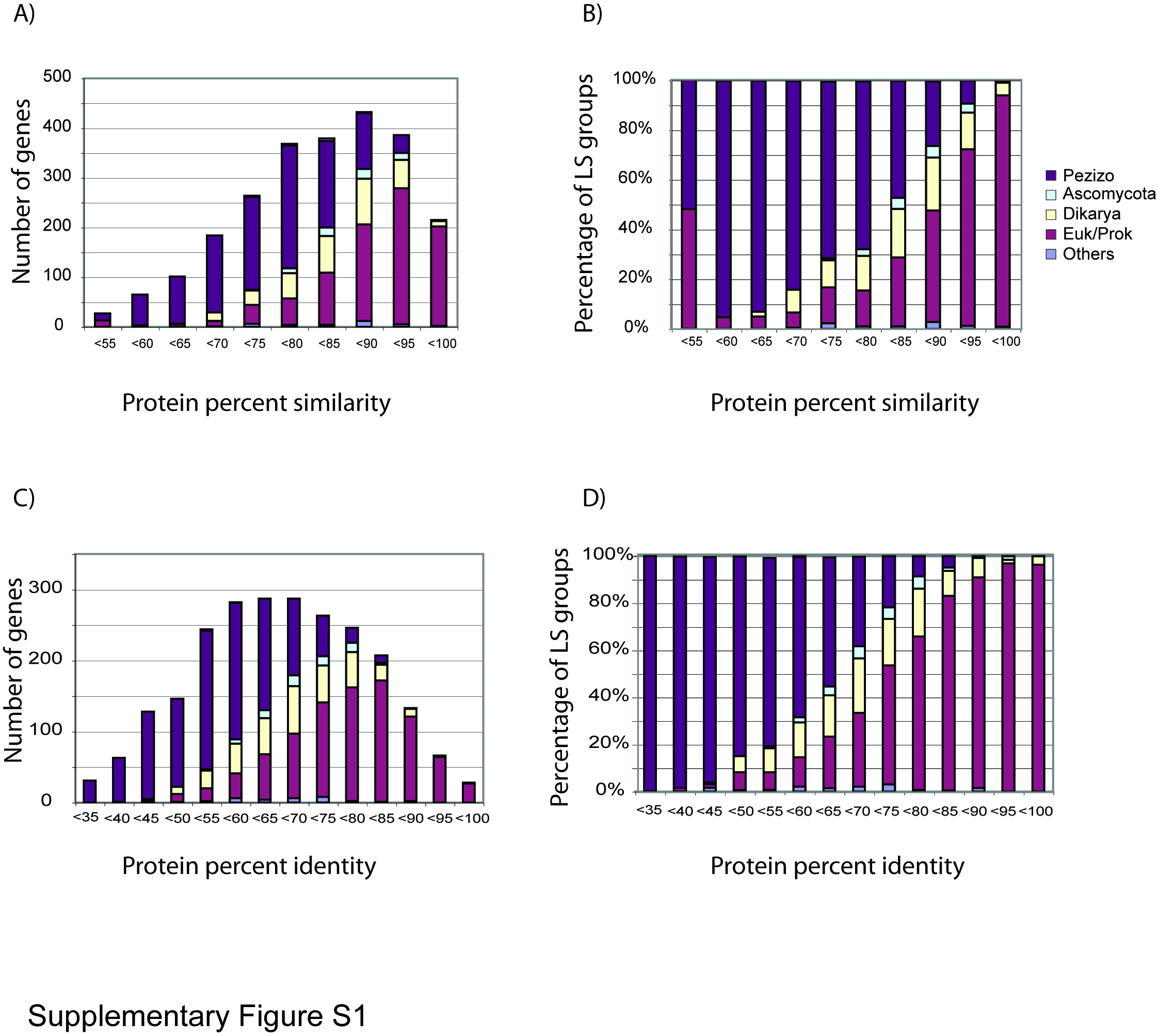

Supplement: Figure S1 — Histograms showing the relationship of percent similarity scores between N. crassa and C. globosum and the number (A) and percentage (B) of PCGs in each of the LS groups. Percent identity scores for number (C) and percentage (D) of PCGs are also shown for comparison. Note that although similarity scores are higher than identity scores, the trend is comparable. (18.59 MB TIF) [file pone.0005286.s003.tif]
